# Dedifferentiated Liposarcoma of the Rectum: A Case Report

**DOI:** 10.1016/j.gastha.2024.03.010

**Published:** 2024-03-22

**Authors:** Kourosh Kalachi, Cheddhi J. Thomas, Paul Savoca

**Affiliations:** 1 Department of Internal Medicine, University of Maryland Medical Center, Baltimore, Maryland; 2 Department of Anatomic Pathology/Neuropathology, Inova Health System, Fairfax, Virginia; 3 Department of Surgery, Inova Health System, Fairfax, Virginia

**Keywords:** Liposarcoma, Dedifferentiated, Rectal tumor

## Abstract

Liposarcoma is a malignant soft tissue tumor that rarely involves the gastrointestinal tract. The dedifferentiated type typically carries the worst prognosis. Here, we describe a rare case of dedifferentiated liposarcoma of the rectum in a young male patient who was treated with surgical excision. Treatment is often difficult and there is no clear consensus on the benefits of radiotherapy or chemotherapy.

## Introduction

Liposarcoma (LPS) is a malignant soft tissue tumor that accounts for a minority of all soft tissue sarcomas. Classified into myxoid, pleomorphic, round cell, well differentiated, and dedifferentiated.^1^ Dedifferentiated LPS (DDLPS) presents as high histologic grade and carries the worst prognosis.^1^ LPS involvement of the gastrointestinal tract is rare, typically involving the stomach, small bowel, and colon.^2^ To our knowledge, there are only a handful of cases in the literature reporting primary LPS of the rectum. Treatment involves surgical resection, although radiotherapy and chemotherapy have been debated.^3^

## Case Report

**Figure 1:**
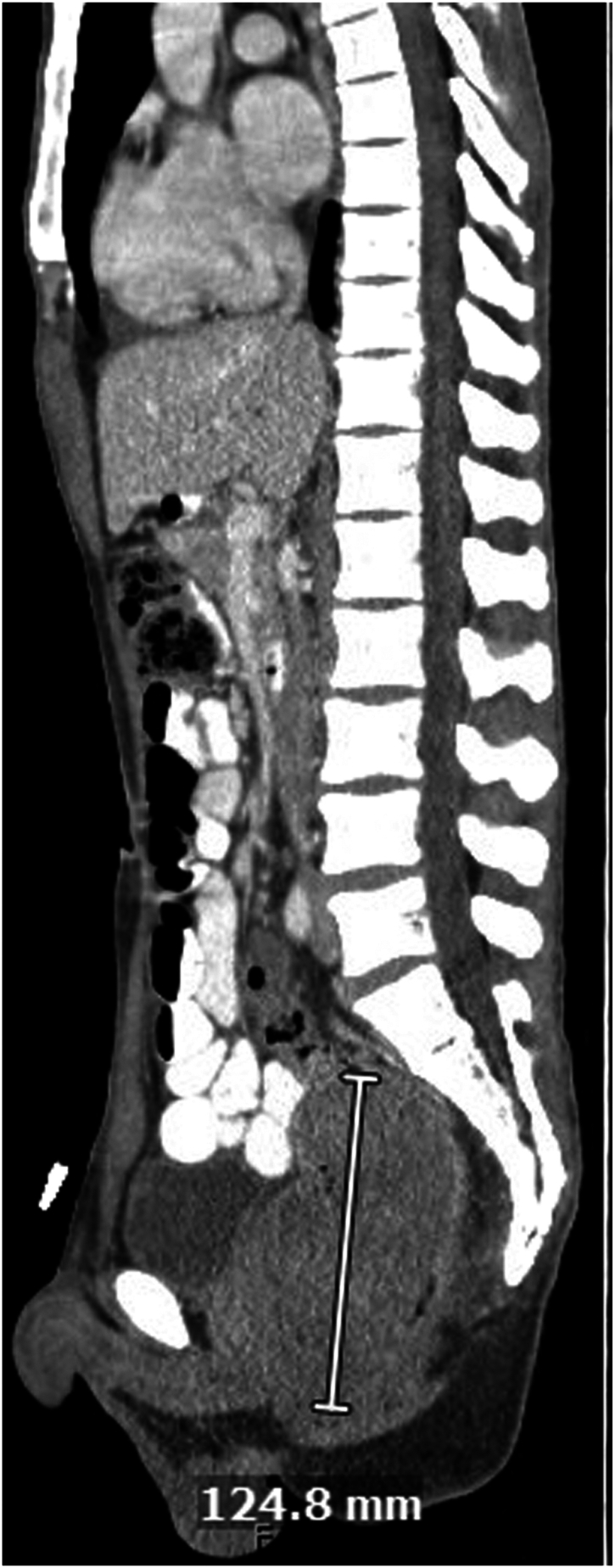
CT scan, coronal view showing large rectal mass.

**Figure 2:**
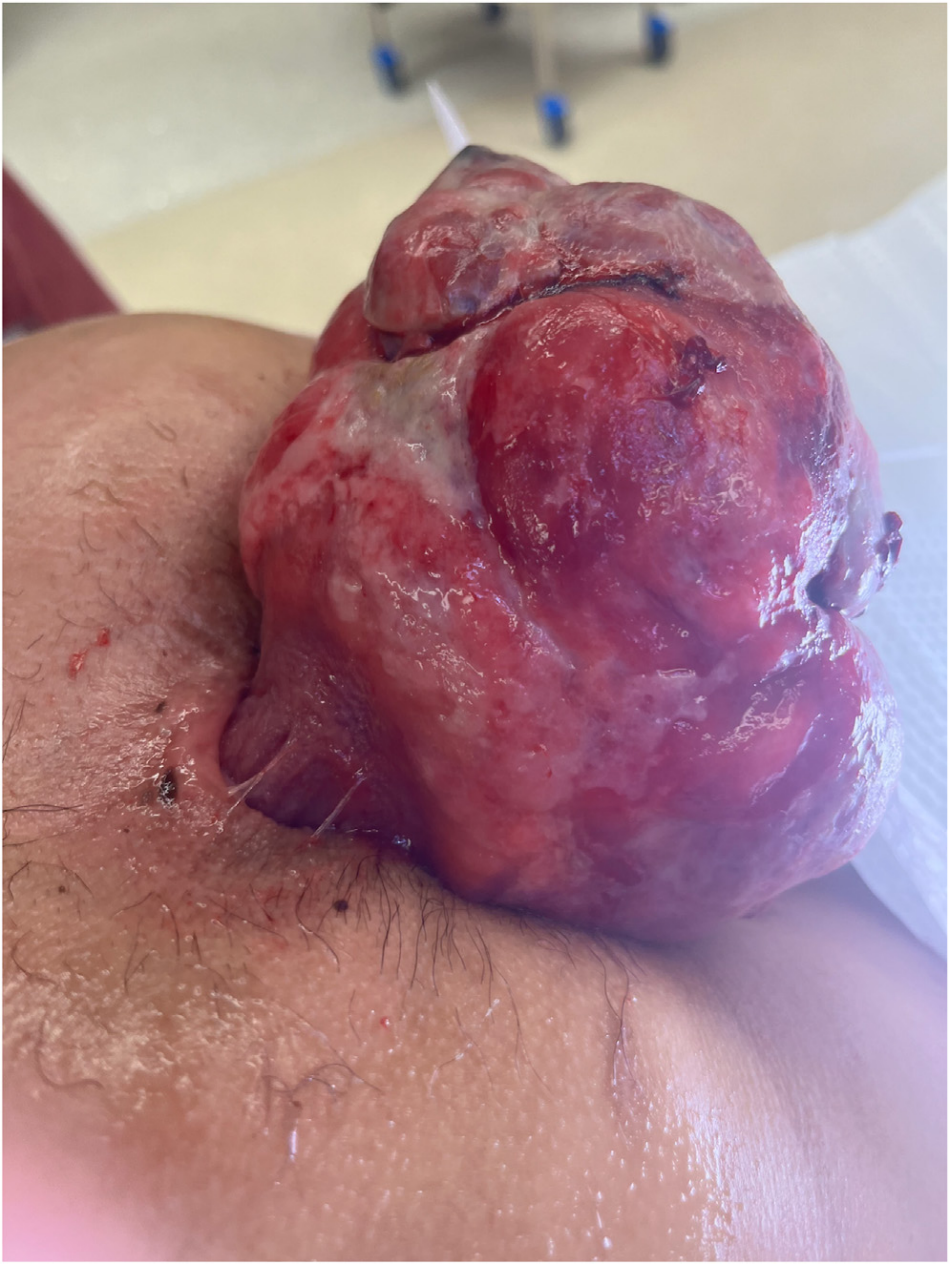
Large, protruding rectal mass discovered on physical examination.

**Figure 3:**
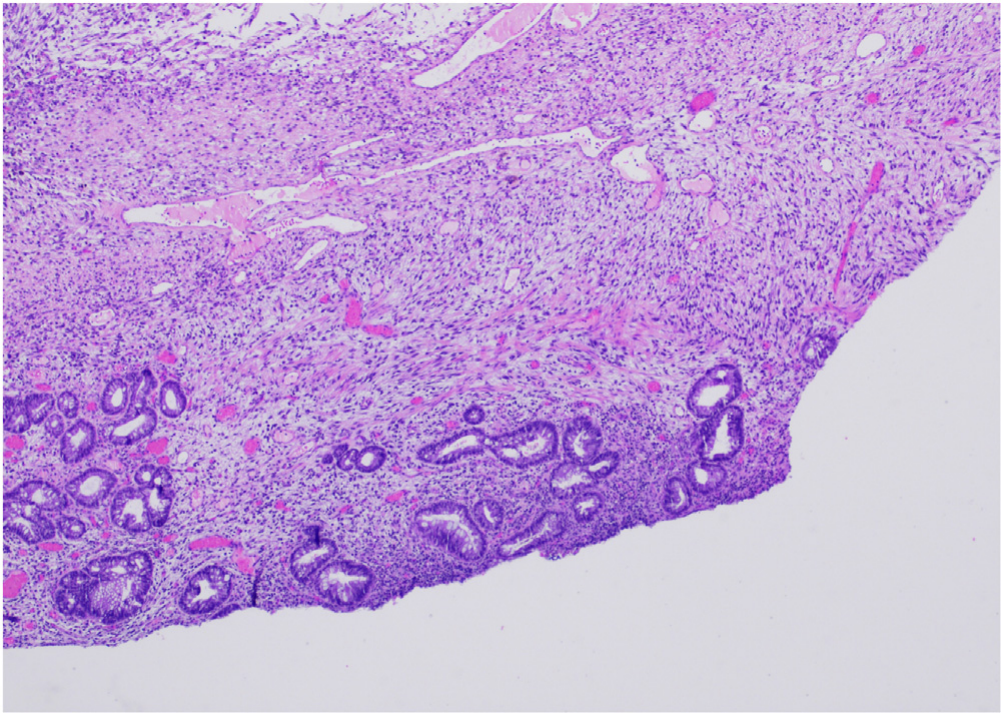
[40X magnification] Spindle cell neoplasm shows transmural involvement of rectum with near complete effacement of microanatomy including the muscularis propria, submucosa, muscularis mucosa, and lamina propria. A focal area of retained lamina propria is seen here.

A 30-year-old Asian male with history of smoking presented with 3 months of worsening suprapubic abdominal pain, loose stools with increased frequency and mucoid discharge, low grade fevers, fatigue, and approximately 10-pound weight loss. He also complained of a fist-sized rectal mass that would intermittently protrude. Initial computed tomography scan showed a 7.1 × 7.4 × 12.5 cm mass in the rectum, without evidence of obstruction or metastasis (Figure 1). Digital rectal examination showed a pedunculated rectal mass at the dentate line, which was extruded under anesthesia at the time of colonoscopy, however unable to be reduced (Figure 2). Colonoscopy to the cecum was otherwise normal. He was transferred directly to the operating room for excision of incarcerated prolapsing rectal tumor. At surgery, the lesion’s 4-cm diameter stalk was amputated and the base originating from the rectal wall over sewn transanally. He was discharged after overnight observation and made an uneventful recovery. Pathology showed a mitotically active spindle cell neoplasm with transmural involvement (H&E 40×) of the rectal wall (Figure 3). By immunohistochemistry, the tumor cells showed strong reactivity for MDM2. The tumor was also positive for CD34 with retained expression of H3K27me3. The tumor cells showed no reactivity for pancytokeratin, beta-catenin, STAT6, S100, Sox10, desmin, CD117, DOG-1, SMA, EMA, Cam5.2, or TLE-1. As this tumor did not demonstrate overt lipomatous morphology, MDM2 reactivity was instrumental in arriving at a diagnosis of DDLPS of the rectum. Following initial resection, he was recommended for trans-anal excision, serial imaging, and proctosigmoidoscopy; however, the rest of his care was transferred to Sloan Kettering where he was recommended for surveillance only and currently doing well 9 months postoperatively.

## Discussion

LPS is a type of soft tissue sarcoma that commonly originates in the extremities and retroperitoneum.^1^ Primary LPS is much less common in the abdominal cavity, with primary LPS of the rectum a very rare occurrence with only a handful of cases reported in the literature. DDLPS of the rectum carries a worse prognosis given its aggressive clinical behavior and high capacity for local recurrence and metastasis, up to 6-fold increased risk of death compared to the well-differentiated histology, with large tumor size (>5 cm) being an adverse independent prognostic factor.^4^ Given its rarity, there are no clear guidelines for treatment; however, surgical resection is currently first line.^5^ The value of perioperative radiotherapy and adjuvant chemotherapy have been debated, and have shown some utility in localized and resectable masses, although DDLPS generally responds poorly to chemotherapeutic agents.^2^ Targeted therapies such as tyrosine kinase inhibitor pazopanib as well as anti-CDK4 and anti-MDM2 oncogenes are emerging and may provide future clinical benefits for the treatment of DDLPS.^6^ Our case shows a rare and unusual presentation of DDLPS in a young male patient.

## References

[1] Tsuruta A. (2012). World J Gastroenterol.

[2] Zheng X. (2023). Medicine (Baltimore).

[3] Li W. (2022). J Int Med Res.

[4] Serafini L. (2020). Dig Dis Sci.

[5] Thway K. (2019). Semin Diagn Pathol.

[6] Gahvari Z. (2020). Curr Treat Options Oncol.

